# Fine Mapping of Two Additive Effect Genes for Awn Development in Rice (*Oryza sativa* L.)

**DOI:** 10.1371/journal.pone.0160792

**Published:** 2016-08-05

**Authors:** Ben Li, Yanpei Zhang, Jinjie Li, Guoxin Yao, Huiqiao Pan, Guanglong Hu, Chao Chen, Hongliang Zhang, Zichao Li

**Affiliations:** Key Laboratory of Crop Heterosis and Utilization of Ministry of Education and Beijing Key Laboratory of Crop Genetic Improvement, China Agricultural University, Beijing 100193, China; Institute of Crop Science, CHINA

## Abstract

Awns, important domestication and agronomic traits in rice (*Oryza sativa* L.), are conferred by polygenes and the environment. Near isogenic line (NIL) pairs BM33 and BM38 were constructed from crosses between awnless *japonica* cv Nipponbare as recurrent parent, and lines SLG or Funingxiaohongmang (awned *japonica* accessions), respectively, as donors. In order to study the genetic and molecular mechanism of awning, two unknown, independent genes with additive effects were identified in a cross between the NILs. To map and clone the two genes, a BC_4_F_4_ population of 8,103 individuals and a BC_4_F_6_ population of 11,206 individuals were constructed. *Awn3-1* was fine mapped to a 101.13 kb genomic region between Indel marker In316 and SNP marker S9-1 on chromosome 3. Nine predicted genes in the interval were annotated in the Rice Annotation Project Database (RAP-DB), and *Os03g0418600* was identified as the most likely candidate for *Awn3-1* through sequence comparisons and RT-PCR assays. *Awn4-2* was fine mapped to a 62.4 kb genomic region flanked by simple sequence repeat (SSR) marker M1126 and Indel maker In73 on chromosome 4L. This region contained the previously reported gene *An-1* that regulates awn development. Thus, *An-1* may be the candidate gene of *Awn4-2*. These results will facilitate cloning of the awn genes and thereby provide an understanding of the molecular basis of awn development.

## Introduction

The domestication of Asian cultivated rice (*Oryza sativa* L.) is a research focus of genetics and archaeology. Common wild rice (*Oryza rufipogon* Griff.) is considered to be the progenitor of cultivated rice [1–3]. A series of morphological and physiological characteristics distinguish the wild and cultivated species, such as seed shattering, stem growth habit, awn length, and hull or seed color [4]. To understand the molecular mechanisms underlying transition of critical domestication traits from wild to cultivated rice, a growing number of genes associated with these phenotypic changes have been identified and characterized by map-based cloning [5–10]; several of them encode transcription factors and have pleiotropic effects [11–14].

The awn that usually has a spinose surface is a spicule-like structure extending from the primordial tip of the lemma [15,16]. Long and burry awns of wild rice are pivotal for propagation since they protect grains against animal predation and facilitate seed dispersal [17]. However, awns in cultivated rice were partially or completely eliminated by artificial selection for the convenience of agricultural practices [18]. Long awns in closed panicles significantly decrease the outcrossing rate [13].

The genetics of awn length and distribution in rice has been studied in intricate detail [18]; many quantitative trait loci (QTLs) were identified in segregating populations developed from crosses between wild and cultivated rice [19–23]. The awn gene *Awn4*.*1* was mapped to a 330 kb region on chromosome 4 using association mapping and linkage analysis [18]. *Awn3-1* was flanked by markers Y5 and Y9 at genetic distances of 0.5 and 0.4 cM on chromosome 3 [24].

Thus far, two major genes for awn length have been cloned using chromosome segment substitution lines (introgression lines) developed from crosses between wild rice (*Oryza rufipogon* Griff.) and *indica* accessions. *An-1*, which encodes a basic helix-loop-helix transcription factor that positively regulates cell division and formation of awn primordia, was mapped to a 70 kb region on chromosome 4. A loss-of-function allele, *an-1*, that increased grain number, underwent strong artificial selection for increased yield [14]. Another cloned gene is *Long and Barbed Awn1* (*LABA1*), mapped to a 34.6 kb region on chromosome 4, and encoding a cytokinin-activating enzyme that positively regulates awn elongation and barb formation [25]. *An-2*, an allele of *LABA1*, promotes extension of awn primordia and decreases grain number per panicle and tiller number per plant, resulting in loss of grain yield [26]. Additional genes affecting awn development differentially expressed between *indica* and *japonica* are characterized [16].

Awn development is a complex trait, usually controlled by multiple genes in rice. In order to avoid the influence of genetic background and interaction between multiple genes for awns, we developed NILs with different single genes controlling awn development and generated segregating populations from crosses between awned *japonica* accessions as donors and awnless *japonica* cv Nipponbare as the recurrent parent. After several generations of backcrossing and self-pollination, recessive and dominant homozygous lines derived from segregating population in a 3 awned: 1 awnless ratio were chosen as NILs. These lines were intercrossed to create populations suitable for gene mapping. This allowed us to predict two candidate genes with additive effect on awn length. The results lay a foundation for study of the molecular mechanism underlying changes in awn development in wild and cultivated rice.

## Materials and Methods

### Plant materials

We constructed the experiment population from crosses between cultivated *japonica* rice cv Nipponbare (awnless) as the recurrent parent and Funingxiaohongmang (*japonica* accessions with long awns) as donor parent. After backcrossing and self-crossing for several generations dominant awned and recessive awnless BC_4_F_4_ individuals were chosen as a BM38 NIL pair from BC_4_F_3_ population probably segregating 3 awned: 1 awnless. The awned line was named BM38a and the awnless sib was named BM38b (S1 Fig). Ten BC_4_F_3_ awned individuals showing 3 awned: 1 awnless segregation ratios were planted for next generation, from which one BC_4_F_4_ segregated population with 180 individuals were chosen for preliminary mapping of the *Awn4-2* gene. The large BC_4_F_6_ population with 11,206 individuals was produced for fine mapping (Table 1).

**Table 1:** Phenotypic and genetic analysis of the BM33 and BM38 NIL lines.

Material name	Population	Dominant(Awned)			Recessive (Awnless)			χ^2^	Theoretical Segregated Ratios
		Plant No.	Frequency of spikelets with awns (%)	Awn length (mm)	Plant No.	Frequency of spikelets with awns (%)	Awn length (mm)		
BM33	BC_4_F_4_	6141	52.85 ± 11.55	29.62 ± 7.58	1962	0	0	1.35	3:1
BM38	BC_4_F_4_	138	70.55 ± 9.85	38.53 ± 8.21	42	7.86 ± 4.23	13.27 ± 6.53	0.14	3:1
BM38	BC_4_F_6_	8351	68.38 ± 8.93	36.47 ± 7.09	2855	7.59 ± 5.81	12.61 ± 7.31	0.68	3:1

In our previous study *Awn3-1* was mapped between markers Y5 and Y9 within a 0.9 cM region on chromosome 3 [24]. To further study and fine map *Awn3-1*, a BM33 NIL pair, awned BM33a and awnless BM33b were selected from a cross between *japonica* rice cv Nipponbare (awnless) as the recurrent parent and SLG (*japonica* accession with long awns) as donor parent (S1 Fig). A BC_4_F_4_ population of 8,103 individuals was developed from 41 BC_4_F_3_ hererozygous dominant awned individuals. The populations were grown in Beijing (N39°, E116°) for phenotyping and mapping.

### Phenotypic Evaluation

Twenty plants of each BC_4_F_4_ or BC_4_F_6_ line were used to measure awn lengths when >1 mm, and percentage of lemmas with an awn. Three main panicles of each plant were collected for phenotyping. The awn length was represented by the average of apical spikelets on all primary branches measured two weeks after heading when awn breakage could be avoided. The proportion of awns was estimated as the percentage of awned spikelets on each panicle, measured at seed maturity. Awnless plants were measured in the same way.

### Allelism tests and analysis of interaction

Allelism tests were used to the verify independence of the two genes; the respective awned and awnless members of the BM33 and BM38 NIL pairs were intercrossed, and the F_1_ and F_2_ populations were genetically characterized. Segregation ratios were evaluated for goodness of fit to theoretical ratios by chi-squared (χ^2^) tests.

### DNA extraction and molecular marker genotyping

Total DNA was extracted from leaves of each plants after heading using the CTAB method [27]. PCR was performed in a 12 μL reaction volume containing 2.0 μL of 10.0 ng/μL template DNA, 1.0 μL of 10× PCR buffer(Mg^2+^), 0.20 μL of 10 pmol/μL dNTPs, 2 μL of 2.0 pmol/μL primer pairs, 0.1 μL of 5.0 U/μL Taq DNA polymerase and 6.7 μL of ddH_2_O. The PCR procedures contained an initial denaturation step (94°C for 5 min), followed by 35 cycles of 94°C for 30 s, 57°C for 30 s, and 72°C for 1 Kb/min. The procedures were completed with an extension step of 72°C for 10 min. PCR products were separated by electrophoresis on 8% naturing polyacrylamide gels at a voltage of 220 V for 1h and visualized by silver staining [28].

### Gene mapping

Contrasting DNA pools, each based on samples from five awned and awnless plants of the BM33 or BM38 NIL pairs, respectively, were prepared for bulked segregant analysis (BSA) [29]. The pools and DNA from Nipponbare were screened with 1,235 SSR primers to identify polymorphic markers. The physical positions of SSR markers were obtained from the Gramene Annotated Nipponbare Sequence 2009 (http://www.gramene.org). Indel and SNP markers were designed using Primer Premier 5 (Premier Biosoft International, Palo Alto, CA) according to sequence alignment of Contrasting DNA pools. The linkage markers in populations were determined, and the genotypes of recombinants were identified.

### Gene annotation and sequencing

Gene annotation was performed with the Rice Annotation Project Database (http://rapdb.dna.affrc.go.jp). Predicted genes were amplified as overlapping fragments from genomic DNA of awned and awnless plants by PCR, and the fragments were sub-cloned to the TA Vector for sequencing. Products were sequenced with an ABI 3730XL DNA Analyzer (TSINGKE Biotech Co. Ltd., Beijing). Sequences were analyzed using DNAMAN software (version 5.2.2, Lynnon Biosoft, Quebec, Canada).

### RT-PCR assays

According to the development of the awn [14], 1–3 cm and 4–6 cm young panicles were obtained and frozen in -80℃ refrigerator. mRNA samples were extracted using Trizol (Invitrogen, Carlsbad, CA, USA) as described by the manufacturer. To disintegrate contamination of genomic DNA, 20 μg RNA were treated with RNase-free DNase I for 30 min at 37°C. First-strand cDNA was reverse transcribed from 2 μg of RNA using SuperScript^™^ⅡReverse Transcriptase (Invitrogen, Carlsbad, CA, USA). RT-PCR was conducted with gene-specific primers. The procedures were 5 min at 95°C followed by 30 cycles of amplification (95°C for 30 s, 55°C for 30 s, and 72°C for 30s) and 72°C extension step for 10 min. The PCR products were identified by electrophoresis on 3% agarose gel electrophoresis.

## Results

### Phenotypic characterization of the BM33 and BM38 NIL pairs

**Fig 1:**
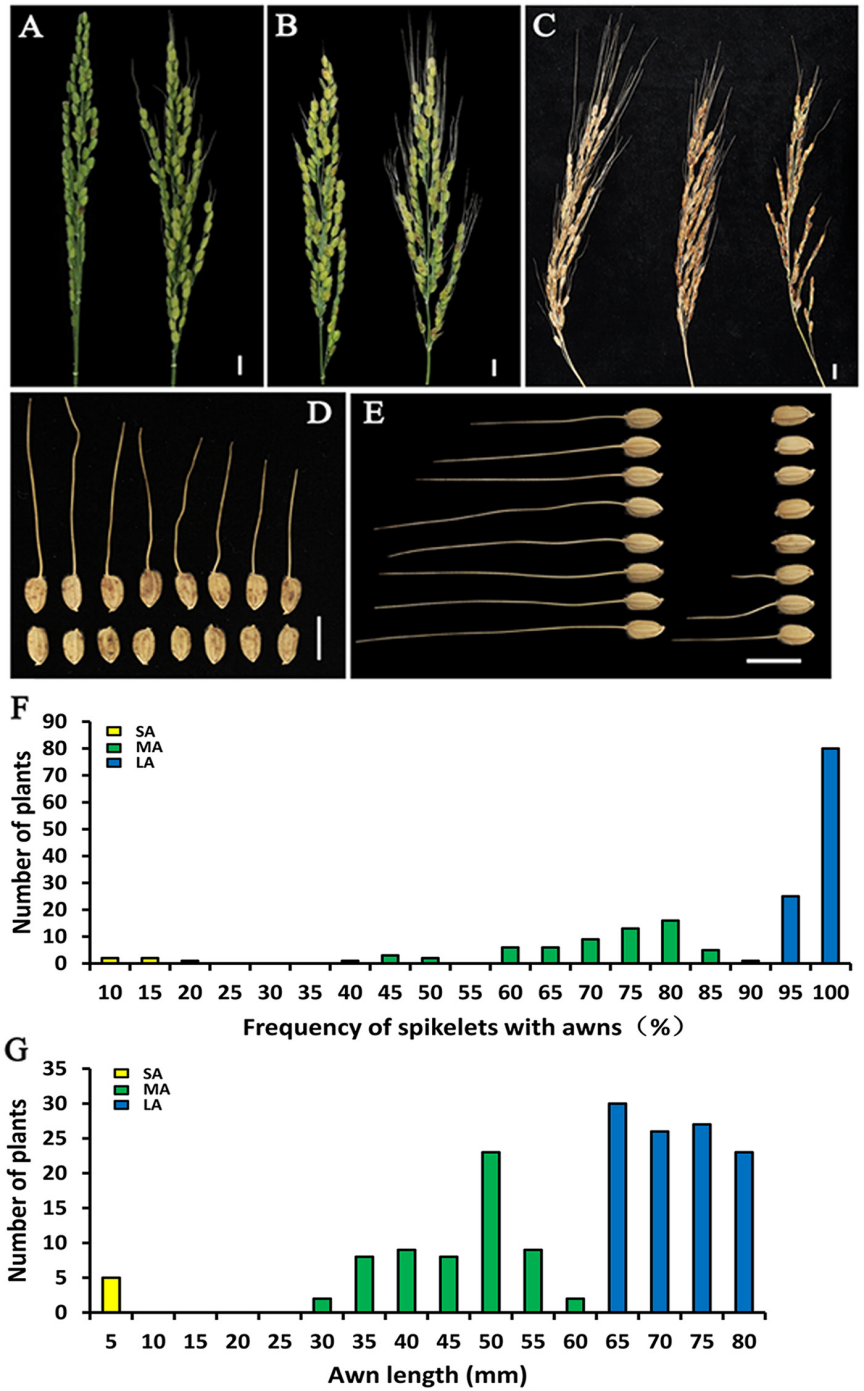
Awn phenotypes. (A) Panicle phenotypes comparing BM33a (right) and BM33b (left); (B) BM38a (right) and BM38b (left). (C) Distinguishable phenotypes in the F_2_ population of the cross between NILs BM33a and BM38a. These classes were consistent with the presence of LA (left), MA (center) and SA (right). Phenotypic comparison of seed arrays from mature spikelets of NIL lines BM33a (upper) and BM33b (lower) (D) and BM38a (left) and BM38b (right) (E). (F) Distribution of awn ratio. (G) Distribution of awn length. Bars, 10 mm. See Table 2 for abbreviations LA, MA, and SA.

The 3 awned: 1 awnless segregation ratios for both populations confirmed single dominant genes for awning (Table 1). The proportion of spikelets with awns for homozygous dominant individuals of BM33a was 52.85 ± 11.55% in single panicle, the awn length was 29.62 ± 7.58 mm, and homozygous recessive individuals of BM33b showed the awnless phenotype. The awn rate for homozygous dominant individuals of BM38a was 70.55 ± 9.85%, the awn length was 38.53 ± 8.21 mm. The awn frequency for homozygous recessive individuals of BM38b was 7.86 ± 4.23%, the awn length was 13.27 ± 6.53 mm. Although the BM33a and BM38a NIL lines were awned their phenotypes were slightly different (Fig 1A, 1B, 1D and 1E). Compared to BM33a, dominant BM38a individuals had longer awns and a higher awn ratio. All of recessive BM33b individuals were awnless, but recessive BM38b individuals had shorter awns and a lower awn ratio (Table 1), which may be caused by different genetic control of awn formation.

### Tests of allelism and effects of two genes

In F_1_ and F_2_ populations of crosses between the BM33b and BM38b lines (both recessive lines) all individuals were awnless. The F_2_ population of the cross between the two awned NILs (dominant lines), BM33a and BM38a, segregated for three different phenotypes (Fig 1C, 1F and 1G). Chi-squared tests confirmed a satisfactory fit to a segregation ratio of 9:6:1 (χ^2^ = 4.15, p>0.05) (Table 2), indicative of genetically independent genes from BM33a and BM38a. From their phenotypes, both the proportion of spikelets with awns and the awn length, we can infer that two independent awn genes have additive effects.

**Table 2:** Segregation of awn phenotype, length and rate in an F_2_ population of the cross between awned NIL lines BM33a and BM38a.

Phenotypic class	No. of individuals	Theretical segeragated ratio	Frequency of spikelets with awns(%)	Awn length (mm)
**Long awn (LA)**	**106**	**9**	**94.63 ± 5.37**	**70.51 ± 7.56**
**Medium long awn (MA)**	**61**	**6**	**60.18 ± 20.63**	**42.57 ± 9.15**
**Short awn(SA)**	**5**	**1**	**8.12 ± 7.33**	**16.42 ± 15.20**

### Physical mapping and analysis of predicted gene *Awn3-1*

**Fig 2:**
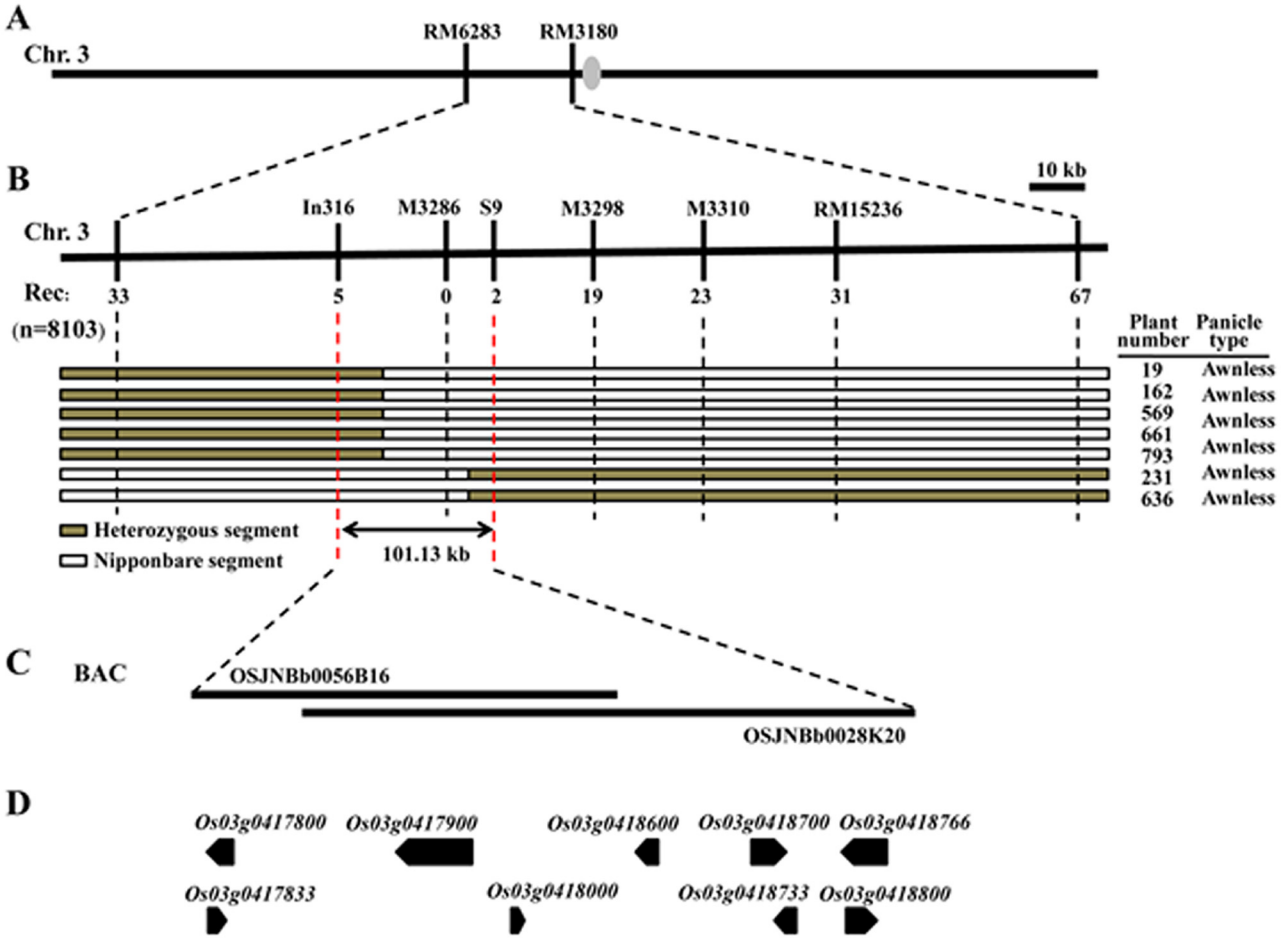
Fine mapping and prediction of *Awn3-1*. (A) *Awn3-1* was located between markers RM6283 and RM3180 on rice chromosome 3. Numbers of recombinants are shown below the position of the eight markers. Chromosomal constitution of eleven recombinants (plant numbers 19, 162, 569, 661, 793, 231 and 636) are shown with their panicle type. Grey and white bars represent chromosomal segment of heterozygous and Nipponbare, respectively. (B) *Awn3-1* was fine mapped to a 101.13 kb genomic region between markers In316 and S9. (C) The Nipponbare genomic BACs (OSJNBb0056b16 and OSJNBb0028K20) in this region. (D) Gene prediction according to RAP-DB. The gray ellipse in (A) represents the centromere.

Morphological characteristics and a previous genetic map showed that the awning gene *Awn3-1* was located within a 0.9 cM region on chromosome 3 [24]. To further map the *Awn3-1* locus, a BC_4_F_4_ population of 8,103 individuals was developed from the cross between SLG (awned *japonica*) and cv Nipponbare (awnless). Two polymorphic SSR markers linked to *Awn3-1* were used to identify recombinants among 1,962 recessive individuals of the BC_4_F_4_ population; 33 and 67 recombinants were found relative to RM6283 and RM3180, respectively (Fig 2A). To narrow the region 51 new primers comprising 20 SSR and 31 Indel were synthesized and tested for polymorphism by bulked segregant analysis [30]. Four SSR markers, RM15236, M3310, M3286, M3298, and one Indel marker In316 were polymorphic and used for chromosome walking. Five of 33 recombinants relative to RM6283 were identified at In316. On the other side of the region, 67 recombinants at RM3180, 31, 23 and 19 individuals at each of RM15236, M3310 and M3298 were recombinant. Two of the 19 recombinants at M3298 were detected using SNP marker S9 obtained by sequencing (S1 Table). From these results, *Awn3-1* was mapped to a 101.13 kb genomic region between markers In316 and S9 (Fig 2B).

To determine the candidate gene for *Awn3-1* the complete mapping region was analyzed according to the Rice Annotation Project Datebase (RAP-DB) (http://rapdb.dna.affrc.go.jp/ version IRGSP-1.0). Nine predicted genes were identified within the region containing two Nipponbare genomic BACs, OSJNBb0056b16 and OSJNBb0028K20 (Table 3, Fig 2C and 2D). It is difficult to perceive a relationship between genes with predicted function and the awn trait. Primers for the nine predicted genes were designed, synthesized, and amplified for sequence analysis using DNA templates from awned and awnless plants (S2 Table). Comparison of the sequences showed no differences between awned and awnless plants.

**Table 3:** Predicted genes in the finely mapped region of *Awn3-1* and *Awn4-2*.

Locus^1^	Predicted function	Size (bp)	cDNA	CDS (bp)
*Os03g0417800*	Nitrilase associated protein-like	3176	AK121654	351
*Os03g0417833*	Hypothetical gene	1229	EU943612	279
*Os03g0417900*	Similar to ARE1-like protein	10350	AK103622	2109
*Os03g0418000*	Similar to basic endochitinase 2 precursor	1043	EU045451	981
*Os03g0418600*	Conserved hypothetical protein	2952	AK122114	477
*Os03g0418700*	Conserved hypothetical protein	4676	AK101797	2778
*Os03g0418733*	Hypothetical gene	1937	AK288673	300
*Os03g0418766*	Hypothetical gene	6105	AK288279	222
*Os03g0418800*	RNA recognition motif domain domain containing protein	4120	AK101685	1572
*Os04g0350700*	Similar to phytochrome-interacting factor 4 (bHLH9)	3599	AK108605	792
*Os04g0351100*	Hypothetical protein	4863	tplb0023j11	435
*Os04g0351333*	Similar to OSIGBa0144C23.4 protein	3122	AK373258	2640

^1^Online source (http://rapdb.dna.affrc.go.jp/ version IRGSP-1.0).

**Fig 3:**
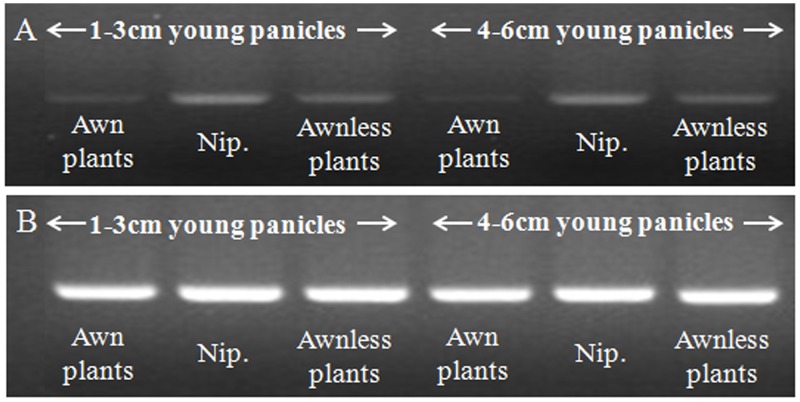
Semi-quantitative analysis of expression of candidate genes in awned and awnless plants, with Nipponbare as a control. (A) Expression of *Os03g0418600*; (B) Expression of *actin1* under the same conditions.

To detect the effects of expression of these predicted genes RT-PCR assays were performed on 1–3 cm and 4–6 cm young panicles of awned and awnless plants as well as Nipponbare. Only *Os03g0418600* showed an expression difference; the level of which was higher in awnless plants than in awned plants (Fig 3). Therefore, the candidate gene of *Awn3-1* was postulated to be *Os03g0418600* with high expression preventing or inhibiting awn development.

### Fine mapping and analysis of predicted gene *Awn4-2*

To locate the awn gene (designed as *Awn4-2*) in the BM38a line 1,235 SSR markers distributed on all 12 rice chromosomes were screened by bulked segregant analysis to identify polymorphisms. Using 180 individuals from the BC_4_F_4_ population developed from the cross Funingxiaohongmang (awned *japonica*) × cv Nipponbare (awnless), *Awn4-2* was flanked by SSR markers RM5687 and M1304 on chromosome 4L with genetic distances of 5.1 and 8.2 cM, respectively. RM742 co-segregated with the *Awn4-2* phenotype.

**Fig 4:**
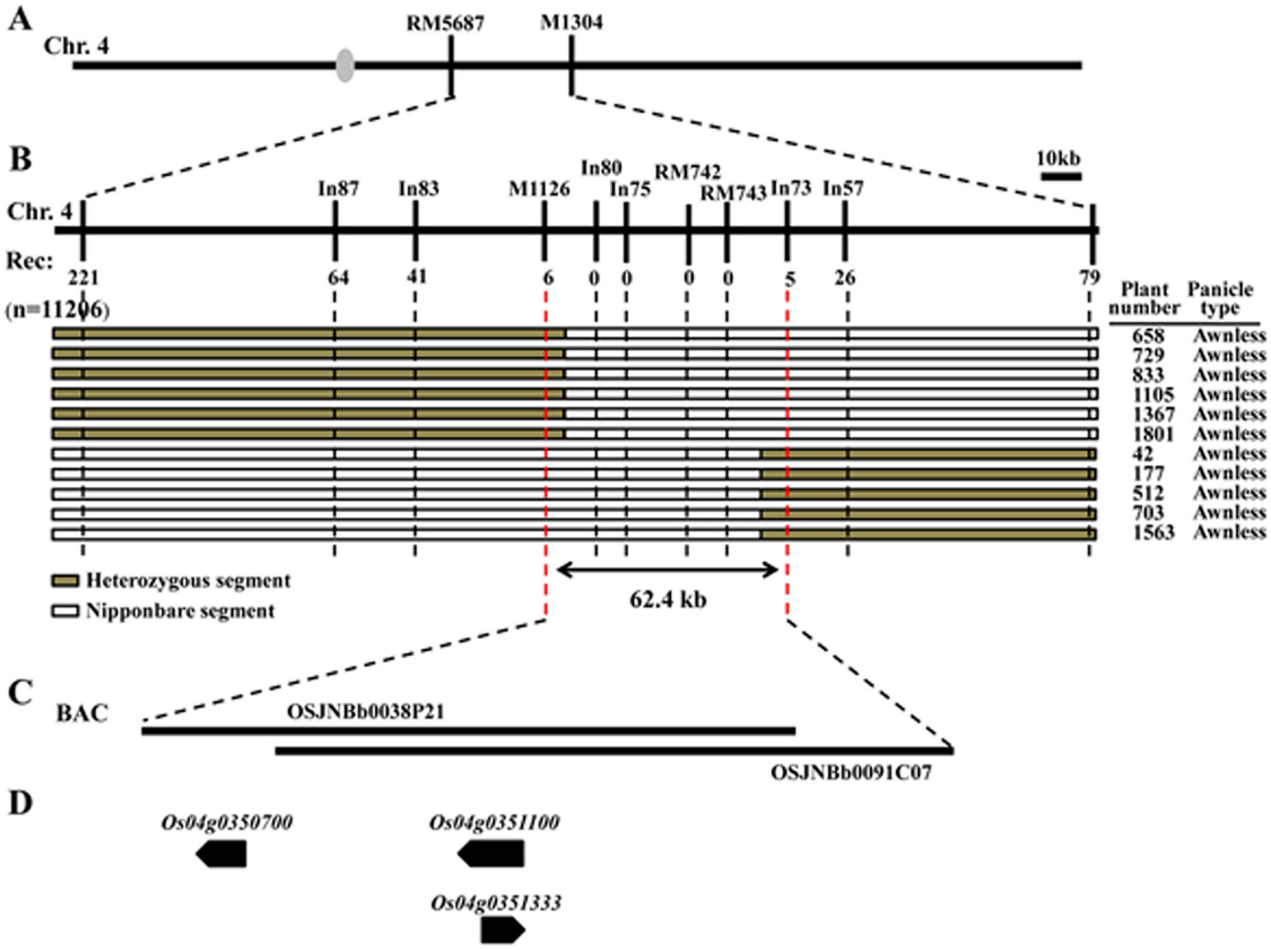
Fine mapping and gene prediction of *Awn4-2*. (A) *Awn4-2* was located between markers RM5687 and M1304 on rice chromosome 4L. Numbers of recombinants are shown below the position of the 11 markers. Chromosomal constitution of seven recombinants (plant numbers 658, 729, 833, 1105, 1367, 1801, 42, 177, 512, 703 and 1563) are shown with their panicle type. Grey and white bars represent chromosomal segment of heterozygous and Nipponbare, respectively. (B) *Awn4-2* was fine mapped between markers M1126 and In73. (C) The Nipponbare genomic BACs (OSJNBb0038P21 and OSJNBb0091C07) in this region. (D) Gene prediction according to RAP-DB. The gray ellipse in (A) represents the centromere.

To refine the position of the *Awn4-2* gene, 33 SSR and 39 Indel markers based on polymorphisms between the *indica* and *japonica* genomes in the primary mapping region were synthesized and screened. One linked SSR marker and four Indel markers (M1126, RM742, RM743, In87, In83, In80, In75, In73, In57 (S3 Table)) were used for fine mapping. Among 11,206 individuals from the BC_4_F_6_ population used to screen for recombinants, 2,855 awnless individuals were genotyped using RM5687 and M1304; 221 and 79 recombinants were identified, respectively. Markers In87, In83, and M1126 identified 64, 41 and 6 heterozygous recombinants among the 221 recombinants relative to RM5687. On the other side, five heterozygotes at In73 were identified among the 79 recombinants at M1304. Finally, *Awn4-2* was mapped to a 62.4 kb genomic region between markers M1126 and In73 (Fig 4B).

**Fig 5:**
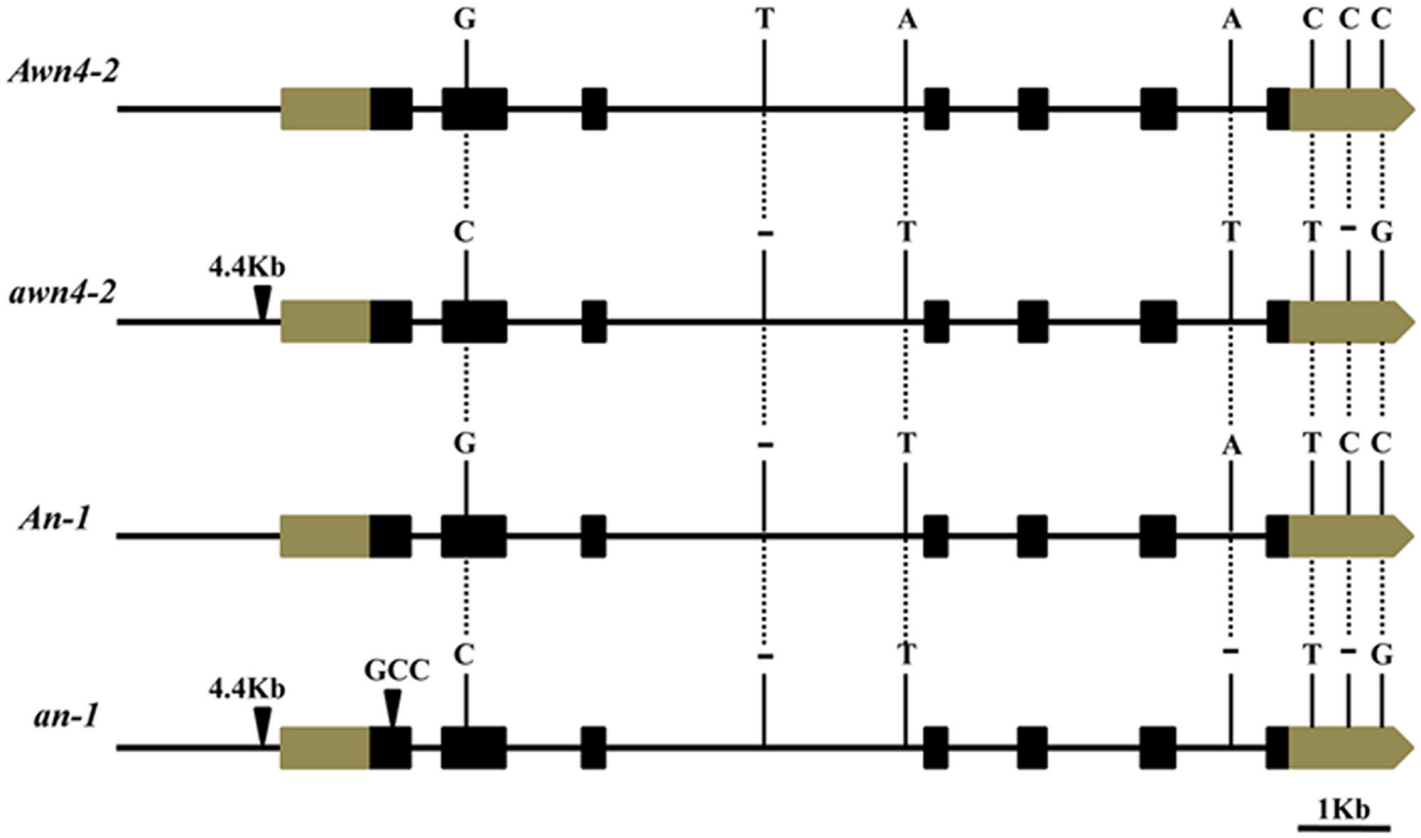
Sequence variation in *Awn4-2*, *awn4-2*, *An-1* and *an-1*. Black bars represent 5’ upstream regions and introns. Grey bars represent 5’ and 3’ untranstated regions. Black boxes represent coding regions. The short dashes represent single base deletions. The black triangles represent a 4.4 Kb or 3-bp insertion. Bar = 1Kb.

**Fig 6:**
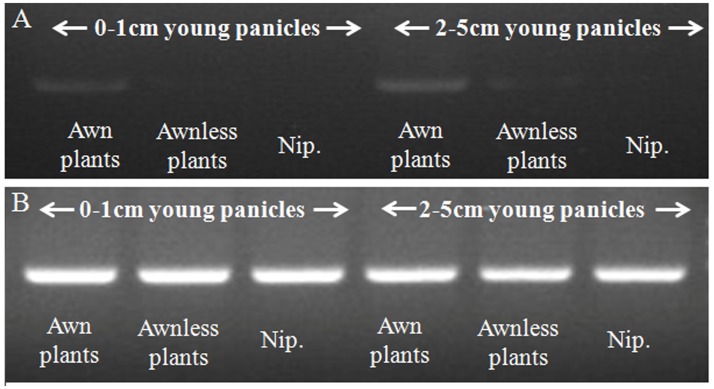
Semi-quantitative analysis of expression of *Os04g0350700*. (A) Expression of *Os04g0350700* in 0–1 cm and 2–5 cm young panicles of awned plants, awnless plants, with Nipponbare as control plants; (B) Expression of actin1 was used as a control.

Three genes were predicted in the 62.4 kb mapped region containing two Nipponbare genomic BACs, OSJNBb0038P21 and OSJNBb0091C07, adopted from the Rice Annotation Project Datebase (RAP-DB) (http://rapdb.dna.affrc.go.jp/ version: IRGSP-1.0) (Table 3, Fig 4C and 4D). To perceive changes in the predicted genes in phenotypically dominant awned and recessive awnless plants primers for the three hypothetical genes were synthesized and amplified for sequence comparison (S4 Table). There were seven mutations (two nucleotide deletions and five nucleotide substitutions) between awned and awnless plants in *Os04g0350700*. This gene was previously reported as *An-1*. A nucleotide substitution (from G to C) was present in the second exon, two nucleotide substitutions (from A to T) and a nucleotide deletion (T) were present in the intron, two nucleotide substitutions (C to T and C to G) and a nucleotide deletion (C) were present in the 3’ UTR region. In addition, *Os04g0350700* of the awnless individuals had a ~4.4 kb inserted fragment in the promoter (Fig 5). According to results of semi-quantitative analysis, *Os04g0350700* showed higher expression in 0–1 cm and 2–5 cm young panicles of awned plants (Fig 6). Based on these results, *Os04g0350700* was postulated to be candidate *Awn4-2* gene.

## Discussion

### The complex genetic mechanism of awn in rice

The awn is a complex morphological trait in rice, and the process of awn development is controlled by multiple genes [18]. When *indica* and *japonica* subspecies accessions are crossed, the inheritance of awns is complex. Segregation of awns was studied in a series of populations derived from a cross between *indica* cv Guangluai 4 (awnless) and *japonica* cv Nipponbare (awnless) [31]. Fifteen QTLs related to the awn phenotype were identified, and laid a foundation for further fine mapping and cloning of the QTL. In this study, mapping populations for studying the genetics of awns were constructed by backcrossing and self-crossing for several generations following an original cross between an awned *japonica* line and awnless cv Nipponbare. This procedure was designed to reduce interference by segregating genetic backgrounds of the original parents, as we wished to simply the inheritance and target single genes for eventual cloning. The strategy proved to be successful and two dominant genes contributing to the awn phenotype were fine mapped and candidate genes were identified. Awn development in rice is also influenced by environment. Late *japonica* rice lines, NK58, NK58s and NK58sr, reportedly showed responses to day length. No awns in panicles were observed under short-day conditions, but under long-day conditions, long awns protruded from the apices of lemmas [32]. The awns of progeny of the cross SLG × Nipponbare grown in Sanya during the present work were shorter than when the same materials were grown in Beijing. We therefore grew our populations for phenotyping in Beijing.

### Interaction of *Awn3-1* and *Awn4-2*

The dominant alleles of *Awn3-1* and *Awn4-2* confer the awned trait. Plants with *awn3-1* and *awn4-2* are awnless or have short awns and low awn ratios (Table 1). An F_2_ population of the cross BM33a ×BM38a NIL lines (*Awn3-1Awn3-1awn4-2awn4-2* × *awn3-1awn3-1Awn4-2Awn4-2*) was classified into three different phenotypes (Table 2): Long awn (LA), medium long awn (MA) and short awn (SA). LA individuals had awns that were longer and with higher awn ratios. SA individuals had no or very short awns and low awn ratios. MA individuals had intermediate awn phenotypes. All individuals derived from the cross between the BM33b and BM38b lines were awnless. These experiments confirmed the presence of two dominant genes with additive effects on awn length and awn ratio.

### A candidate gene for *Awn3-1*

High expression of *Os03g0418600* occurred in awnless plants (Fig 3); this gene encoded a conserved hypothetical protein was presumed to be the candidate for *Awn3-1*. Many genes positively affect awn phenotype in rice, including *An-1* [14]. *DROOPING LEAF* (*DL*) located on chromosome 3 affected awn development together with gene *OsETTIN2* (*OsETT2*) in *indica* cv Kasalath, with reduced expression leading to less awn development [16], which was opposed to *Os03g0418600*. However, decreased expression of *TONGARI-BOUSHI1* (*TOB1*) led to awn elongation, a similar mode to that of *Os03g0418600*, but there was no evidence of homology between them. If the presumed function of *Os03g0418600* is confirmed by gene knockout, this may be a new control path for rice.

### *Awn4-2* is a new allele of *An-1*

*An-1* was identified as a major awn QTL with an important role in rice domestication. The parents were one accession of *Oryza rufipogon*, W1943 (long awn) and *indica* cv GLA4 (awnless) [14]. In the present study, *awn4-2* plants showed an awn proportion of 7.86 ± 4.23% (Table 1). Sequence differences between *An-1* and *an-1* were compared with differences between *Awn4-2* and *awn4-2*. A 3 bp insertion in the first exon of the *an-1* did not occur both *Awn4-2* and *awn4-2*, whereas three SNPs (two A to T, one C to T) were found between *Awn4-2* and *awn4-2* and not between *An-1* and *an-1*(Fig 5). Hence, *Awn4-2* appears to be a new haplotype of *An-1*. The differences of awn length between *an-1* and *awn4-2* plants may be caused by the 3 bp deletion/insertion in the first exon and a nucleotide variation (T/-) in the sixth intron.

Distribution and length of awns in rice are sensitive to genetic background, but the mapping populations of *Awn3-1* and *Awn4-2* whose genetic backgrounds were *japonica* were suitable for identifying interaction between them. Preliminary study found that *Awn3-1* and *Awn4-2* have additive effects on awn length and proportion lemmas per spikelet with awns. The underlying molecular mechanisms might be revealed following cloning.

## Supporting Information

S1 Fig Graphic genotype of NILs BM33 and BM38. White regions represent the Nipponbare genotype. Black regions represent the SLG genotype. Grey regions represent the Funingxiaohongmang genotype.
(TIF)

S1 Table Markers and primers used in physical mapping of *Awn3-1*. (DOCX)

S2 Table Sequencing primer pairs used for predicting candidate gene *Awn3-1*. (DOCX)

S3 Table Markers and primers used for primary and fine mapping of *Awn4-2*. (DOCX)

S4 Table Sequences of primers used for predicting the *Awn4-2* candidate gene. (DOCX)
