# Supplementary figures and images for: Fine Mapping of Two Additive Effect Genes for Awn Development in Rice (Oryza sativa L.)
Source: PLoS One. 2016 Aug 5;11(8):e0160792. doi: 10.1371/journal.pone.0160792 (PMC4975416; doi:10.1371/journal.pone.0160792)

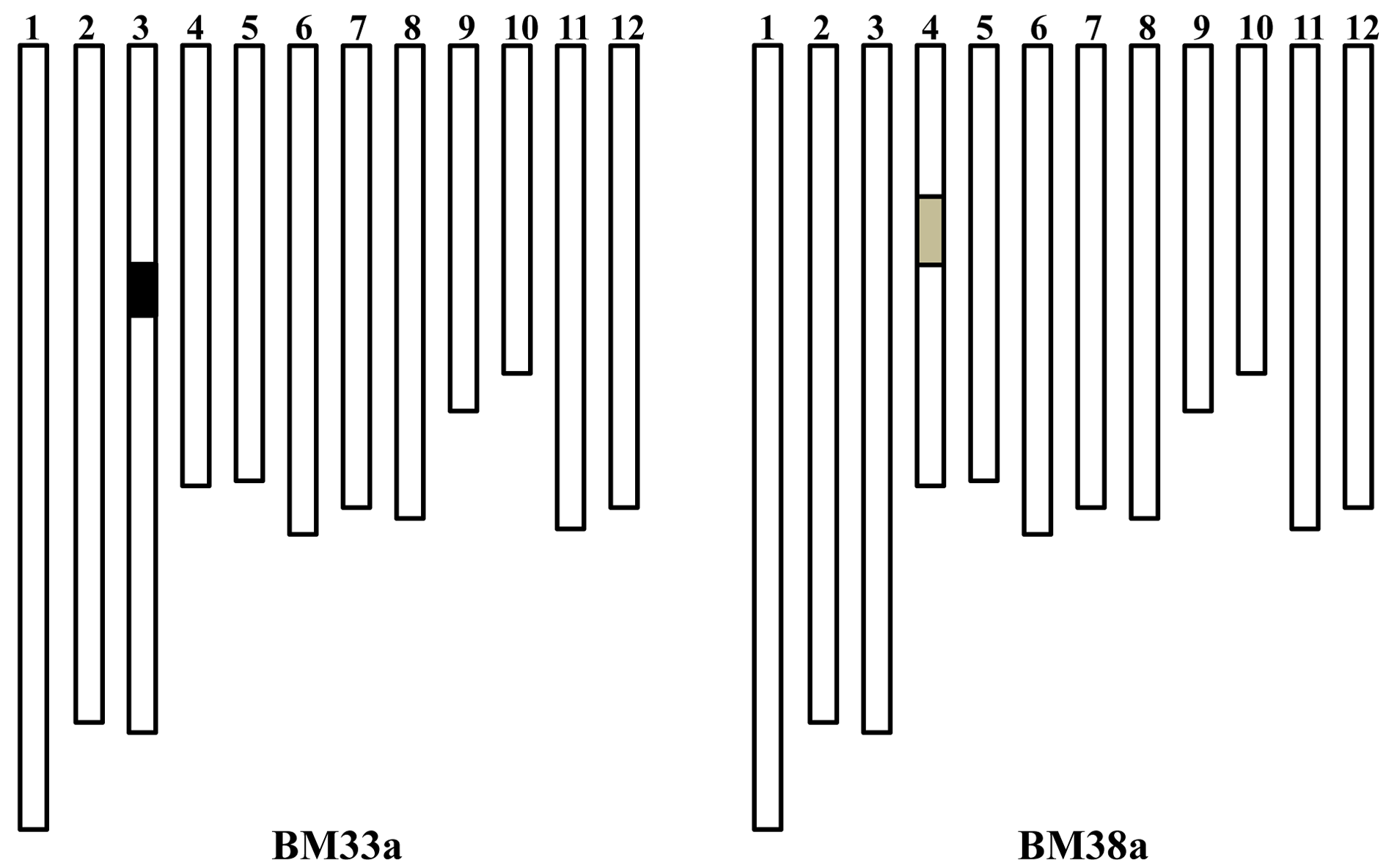

Supplement: S1 Fig — White regions represent the Nipponbare genotype. Black regions represent the SLG genotype. Grey regions represent the Funingxiaohongmang genotype. (TIF) [file pone.0160792.s001.tif]
