# Supplementary material for: Fine Mapping of Two Additive Effect Genes for Awn Development in Rice (Oryza sativa L.)
Source: PLoS One. 2016 Aug 5;11(8):e0160792. doi: 10.1371/journal.pone.0160792 (PMC4975416; doi:10.1371/journal.pone.0160792)
Supplement: S1 Table — (DOCX) [file pone.0160792.s002.docx]

**S1 Table. Markers and primers used in physical mapping of *Awn3-1***

| **Marker** | **Forward primer（5’ to 3’）** | **Reverse primer（5’ to 3’）** |
| --- | --- | --- |
| RM6283 | GATCAGGTGGTCGGTTCCTTA | CCTGTTGGAGACTGAGCTGAT |
| In316 | ACTCACTTCTGAGCCCCAAG | TTGGGATAGAAATCAGTTAAA |
| M3286 | CTTTGTGAAAGCTTCCCTGC | CAAAGCTATGCTCCTCCGTC |
| S9 | CAAACTCTATCCGATGTCTCA | ACTTGAAATGTTCCCGTTAT |
| M3298 | CCTTCTTGGGAACCATCAAA | TTACGCACATCCTCTTTCCC |
| M3310 | CATTTAGAGGCCGGGATTC | AAAAGGAGAGAGGAGGACGC |
| RM15236 | CACTCCCTCCTCTCTCCTCTCC | GTTGGTTGGTCGGTTGCTTACC |
| RM3180 | GGGTCGGATAGCCACACAC | GAGGTAATCTCGCGGAGTTG |
