# Supplementary material for: Fine Mapping of Two Additive Effect Genes for Awn Development in Rice (Oryza sativa L.)
Source: PLoS One. 2016 Aug 5;11(8):e0160792. doi: 10.1371/journal.pone.0160792 (PMC4975416; doi:10.1371/journal.pone.0160792)
Supplement: S2 Table — (DOCX) [file pone.0160792.s003.docx]

**S2 Table. Sequencing primer pairs used for predicting candidate gene *Awn3-1***

| **Primer name** | **Forward primer（5’ to 3’）** | **Reverse primer（5’ to 3’）** |
| --- | --- | --- |
| 378-1 | GTCCAGGTAACACCCATTTCCT | ATAGAGCTGATGGCCAGAACAC |
| 378-2 | TTTGACTAACTGTTGGCCAGAAC | CTGCATGCTTGCTGTGTTCTATT |
| 378-3 | GCACGCAAGTGTATATGCTTCT | CTCCCCCATTTCGAACGGAT |
| 378-4 | CAGGAAGATCCGGGCCACTC | AATCACTTTGGCACGAGGATCA |
| 379-1 | CCGTTATTGCGGTTAAGGGATA | GATTACGAAGCCAATCAGAGGC |
| 379-2 | CCAGTAGAAGTTCCTCCTGCAT | GCGAATCCCATGTTTGGACTCT |
| 379-3 | TAACCTGTGCAAGTGTGCAATG | GGATGTGACGGGACACTCATAA |
| 379-4 | TTCCTACGGGTAAAGGACGATG | CATGCCACGAGACACCTATCAT |
| 379-5 | GACAGGACTAAGTGTCTGGCAA | ATATATGCGGAAGTTCGTGCAG |
| 379-6 | CTCAAGCATGCCTGCTACTACT | AGTCACATCAATGTCCCTCTGG |
| 379-7 | CAGCAAGATGCCATGAATCCAG | GAGTCCTTGGCAAGAGATCCAA |
| 379-8 | AGTTAAACTGAACATTCTCACG | TCTCAAGAGTAGATGCCAGAGC |
| 379-9 | CACATGGCTGTTCCTTCGGTAA | ACGAATCCATCCAATGACTTGC |
| 379-10 | TCCTACAAAGAGGGAACACTAC | AACTTGAAAGCCCAGCCCAATA |
| 380-1 | CAAGATGCCAATACTACGCCCA | CGTCGTGCATTACTGATCATCG |
| 386-1 | TTTCACTCCTTGCTGCAAATCG | CTGCAGCCATATACGCACAAAT |
| 386-2 | ACTCAGACCAATAACTCAGCTC | GCTGATCTTCTTCCCAGGACAA |
| 386-3 | AATGTCGCATCCCAATGTTTGC | GACTCTAACCTAGCAGACACGC |
| 386-4 | AGGGGATGAGGAATGTGGGA | CTTGATTCATCGGTGGCGTTTT |
| 386-5 | GGCTGCACTAGTACAAAACACG | CATGTGTGCAGTGCTAAAGCTA |
| 386-6 | TTAGATTGGCTTTGTTCGCCG | GAGCCTAACCATCCTAGGGTTT |
| 387-1 | GCATTCCTTCCTTAACACTGGC | GTGCAATTGCCCATTACTACAAC |
| 387-2 | TTAGAAAGTACTGCTGCTGCCA | TAGCCAGAGTTCTTCAGACGTG |
| 387-3 | CAAGACAGCAAGAAGCCTTCAG | TCACGATTGGGTGTTGAAGCTA |
| 387-4 | CGCAAACAAAGAGCAACTCTGA | TAATCGGGTAGCTTGATGGTCG |
| 387-5 | TGTTGAGTGAGGAAGCTCTGAA | CTGATGGCACATGAGCATTTCT |
| 387-6 | TTTGCCCTAACCCTCGATTT | GGGACCTAAGATGAACTTATTCC |
| 388-1 | CCCTTCACCACTTTCCCTCC | AGAAGGCATACGCTCATTAGGC |
| 388-2 | TGCTATGTGATGTGTCCTGATG | GACGGCTCATACGGAAACCAAT |
| 388-3 | CGGTGTTCCCTCCTATCTGTTT | GCCTACCAGACAGTTGACAAGA |
| 388-4 | TTCTTTCTTCAGCTATGGCAGTG | AGCATCGTTGTTATCATTCGGAG |
| 388-5 | GTTGTCAAACCTAAGCCAGCTC | CAGCATTCCCATTATCACCACG |
| 388-6 | ACTGTCTGGTTTGTTGAGTCAT | ACTTCTCCATCATTCCACCCTG |
