# Supplementary material for: Fine Mapping of Two Additive Effect Genes for Awn Development in Rice (Oryza sativa L.)
Source: PLoS One. 2016 Aug 5;11(8):e0160792. doi: 10.1371/journal.pone.0160792 (PMC4975416; doi:10.1371/journal.pone.0160792)
Supplement: S3 Table — (DOCX) [file pone.0160792.s004.docx]

**S3 Table. Table S3 Markers and primers used for primary and fine mapping of *Awn4-2***

| **Markers** | **Forward Primer（5’ to 3’）** | **Reverse Primer（5’ to 3’）** |
| --- | --- | --- |
| M1304 | GGAGTGCGTGCCTCTATCTC | TTTTATGCGCGACTTGTTTTT |
| RM5687 | GATCGCTGGCGATTGATC | GACTTGTGGGGTGGTTTTTG |
| In57 | TTCCCGATCTCCTTCTCCT | TACGACGACCTCCCAATG |
| In73 | TGTAAACTATGATAAAAAGTC | ATTTTATACTGATTCTCCATCGT |
| RM742 | GAACAGAATCCAGGAATGAA | GTCAGATCAGTCTTCTGCAAAT |
| RM743 | TAAATGCCACTTGGCAGTCTAG | TATCTGCACACTGCACTTCAACG |
| In75 | TAAAACCAACATTTCTAACGC | CCTGCTAGACACTCCTGCTA |
| In80 | GACGGCCCTTTCAAAGTATCC | ATGGGTGTGCAAGGCAATCA |
| M1126 | TGATTTTTGGGTTCATTCATCA | ATGGGACGGAGGGATTAACT |
| In83 | TTCTCTCGATAAATGGTTGG | GACAAGCGTGTCAAGTTCTA |
| In87 | TGTCATGTCCCTTCTCATCT | GTACAATGCAGATCCTGGAC |
