# Supplementary material for: Fine Mapping of Two Additive Effect Genes for Awn Development in Rice (Oryza sativa L.)
Source: PLoS One. 2016 Aug 5;11(8):e0160792. doi: 10.1371/journal.pone.0160792 (PMC4975416; doi:10.1371/journal.pone.0160792)
Supplement: S4 Table — (DOCX) [file pone.0160792.s005.docx]

**S4 Table. Sequences of primers used for predicting the *Awn4-2* candidate gene**

| **Primer name** | **Forward Primer（5’ to 3’）** | **Reverse Primer（5’ to 3’）** |
| --- | --- | --- |
| B42G1-1 | GTTGGAAATGCCAATGCTGGTA | CTCTCCTTCTGTACATACGCCC |
| B42G1-2 | TGTAGGATCAAGCCAAAGCCAT | CGGTGGTCAAATTTGGGTTGAA |
| B42G1-3 | ACTGTGAGACACACCAATGTCA | CCACCGATAGCCATAGCCTC |
| B42G1-4 | GGACGAGGAGGTGGAGAAGTAG | ATGTCAAGATGCCAGGAAGGCC |
| B42G1-5 | GCAATTAGTAACACGGTGCG | CGATGGCCGACGAGCACTT |
| B42G2-1 | TATGTATTCTACGACGCAGGGC | GGGAATAAGGGTGGTCACCTG |
| B42G2-2 | CCGCAGGTGACCACCCTTAT | TATCGTGTCCGGGTGCTCAA |
| B42G2-3 | AGCCGCCAAAGATCAGGAAG | CCTCTTCTTGCTCATCACGC |
| B42G2-4 | CGACCTCGTTGCCGTCCTTT | CGCCCTGCTCCATCATCTCA |
| B42G2-5 | CAACATCCTCATCGACTGCTACC | ATAGCCATCAACAAGCGTACCA |
| B42G2-6 | CAAAGAGGGAAGGGTAGCAGAA | AATGCATACACCTACCTGTGCT |
